# Circular RNAs and their roles in idiopathic pulmonary fibrosis

**DOI:** 10.1186/s12931-024-02716-2

**Published:** 2024-02-06

**Authors:** Akshaya Surendran, Chaoqun Huang, Lin Liu

**Affiliations:** 1 The Lundberg-Kienlen Lung Biology and Toxicology Laboratory, Department of Physiological Sciences, Oklahoma State University, 264 McElroy Hall, Stillwater, OK 74078 USA; 2 Oklahoma Center for Respiratory and Infectious Diseases, Oklahoma State University, Stillwater, Oklahoma USA

**Keywords:** Circular RNA, Idiopathic pulmonary fibrosis, Fibroblasts, Alveolar epithelium

## Abstract

Idiopathic pulmonary fibrosis (IPF) is a progressive and fatal lung disease with limited treatment options. Circular RNAs (circRNAs) have emerged as a novel class of non-coding RNAs with diverse functions in cellular processes. This review paper aims to explore the potential involvement of circRNAs in the pathogenesis of IPF and their diagnostic and therapeutic implications. We begin by providing an overview of the epidemiology and risk factors associated with IPF, followed by a discussion of the pathophysiology underlying this complex disease. Subsequently, we delve into the history, types, biogenesis, and functions of circRNAs and then emphasize their regulatory roles in the pathogenesis of IPF. Furthermore, we examine the current methodologies for detecting circRNAs and explore their diagnostic applications in IPF. Finally, we discuss the potential utility of circRNAs in the treatment of IPF. In conclusion, circRNAs hold great promise as novel biomarkers and therapeutic targets in the management of IPF.

## Introuction

Idiopathic pulmonary fibrosis (IPF) is a chronic, progressive age-related fibrotic interstitial lung disorder. The disease is mostly irreversible and is considered to impose a significant health burden on the population due to its high mortality rate and reduced quality of life. It is mostly diagnosed in elderly adults ranging from 50 to 85 years age and 54% of them are males [1, 2]. Etiology is unknown although several risk factors have been identified. The median survivability is 2–5 years after the diagnosis. The prevalence of IPF has been increasing in the last few years. Its impact on the quality of life of patients, poor prognosis, diagnostic challenges and need of effective therapeutics makes this disease a significant health burden across the globe. Early and accurate diagnosis coupled with appropriate and economically feasible treatment options are very crucial when dealing with such conditions.

Circular RNAs (circRNAs) are long non-coding RNA molecules which can be found in a vast variety of living organisms. They are unique by having a ring structure formed by a covalently closed bond and lacking poly-A tail and cap unlike other RNA molecules [3]. The majority of circRNAs originate from exon regions of the pre-mRNA. Interestingly, there are few less common circRNAs which can arise from intron sequences, intergenic genomic regions, 3’ untranslated regions (UTR) and 5’ UTR [4]. They have high stability and are more resistant to RNase R than linear mRNAs [5]. They interact with non-coding RNAs such as microRNAs (miRNA) and proteins to exert biological functions. Many studies have uncovered regulatory mechanisms involving circRNAs in the pathogenesis of numerous diseases such as cancer, diabetes mellitus and neurological disorders [6, 7]. However, limited, but increasing studies have linked circRNAs to IPF. Here, we briefly introduce IPF and circRNAs and discuss the major regulatory and functional roles of several circRNAs in various lung fibrosis models in a detailed manner, followed by potential application and constraints of employing circRNAs in the diagnosis and treatment of IPF.

## Idiopathic pulmonary fibrosis

### Clinical symptoms and current managements

In IPF, the healthy lung tissue is replaced by abnormal fibrous tissue, resulting in altered lung architecture, improper gas exchange and ultimately respiratory failure [8]. Clinically, IPF is a diagnosis of exclusion and is usually done by examining radiographic or histopathologic pattern of usual interstitial pneumonia (UIP). UIP can be defined by the presence of sub-pleural cystic spaces that are often referred as “honey combing”, dilatation of the bronchi and peripheral alveolar wall thickening [9].

Patients with IPF exhibit severe exertional dyspnea with dry cough. Several studies have found associations between the severeness of dyspnea and survival in IPF patients. Cough is the most prominent problematic phenotypic feature of IPF [10–13]. On auscultation of the posterior lung lobe, fine crackles or Velcro rale like crackles can be heard especially during inspiration. Additionally, 30–50% of patients report having clubbed fingers. Other clinical signs include emaciation, hemoptysis and exercise intolerance.

The primary treatment for IPF is lung transplantation, but only a limited number of patients can receive this treatment due to the scarcity of donors and the risk of allograft rejection [14]. Non-pharmacological treatment of IPF includes pulmonary rehabilitation. The pharmacological treatment mainly involves two antifibrotic drugs: nintedanib (an intracellular tyrosine kinase inhibitor) and pirfenidone (an antifibrotic and antioxidant molecule), supported by other therapies such as antibiotics and antacids [15, 16]. These antifibrotic drugs have improved the condition in moderately advanced IPF patients by improving forced vital capacity, and slowed the progression of the disease, but do not cure the disease [17]. The current goal of IPF management is to alleviate symptoms, enhance the quality of life and retain the lung function [18].

### Epidemiology and risk factors

#### Epidemiology

It is essential to understand the occurrence of IPF globally and country-wise to grasp its health and economic burden on the population. But accurate data depicting the incidence and prevalence of IPF is very limited [19]. Moreover, risk factors such as sex [20], smoking [21], dust inhalation [22] and genetic factors [23, 24] can cause heterogeneity in the incidence and prevalence which makes epidemiological studies much harder. Globally, the adjusted incidence ranges from 0.09 to 1.3 per 10,000 persons whereas the adjusted prevalence ranges between 0.33 and 4.51 per 100,000 persons. While comparing the latest data with previously available ones, it is evident that the incidence and prevalence of IPF has increased substantially over years and it is high in countries such as South Korea, Canada, and US [25]. A study conducted from 2010 to 2019 among U.S veterans, the prevalence of IPF has increased from 276 cases to 725 cases per 100,000 persons indicating that the annual incidence increases from 73 cases to 210 cases per 100,000 persons [26].

#### Risk factors

As the name suggests, the etiology of IPF is unknown. However, it is believed that environmental factors, genetics, aging, and microorganisms contribute to the onset of IPF.

##### Aging

Aging is considered as the most significant risk factor in IPF. Aging contributes to the pathogenesis of IPF by impairing progenitor cell renewal, which prevents alveolar epithelial cells from healing and replacing damaged lungs. In IPF, alveolar epithelial type II cells (AEC2) exhibit the traits of aging such as genomic instability, telomere attrition, cellular senescence, stem cell exhaustion along with loss of proteostasis, mitochondrial dysfunction, and altered intercellular communication [27]. AEC2 of IPF patients has shown a high level of dysfunctional mitochondria [28, 29]. Moreover, as the age progresses, genetic damage can build up since the DNA repair can be severely altered with the age progression [30]. This alteration in the DNA repair and genetic instability can lead to cell death. A causal link between loss of AEC2 and development of IPF has been suggested in a study, where the targeted deletion of AEC2 has led to the development of pulmonary fibrosis in mice [31]. Aging also causes several epigenetic changes constituting of DNA methylation, chromatin remodelling, loss of histones, and dysregulation of miRNAs, leading to abnormal alteration of the lung epithelium [1, 32].

##### Environmental factors

Environmental exposures such as air pollution, cigarette smoking and inhalation of wood, metal or silica dust have been considered as major risk factors for the development and progression of IPF, since they can cause injury to a genetically susceptible lung [33]. Among these environmental factors, chronic cigarette smoking provokes a certain epigenetic reprogramming in the human genome through the alteration in DNA methylation [34]. It also induces ER stress, mitochondrial dysfunction, and imbalances in miRNAs, thereby inducing epithelial injury [28, 35, 36].

##### Genetics

IPF with respect to genetics can be classified as familial and sporadic IPF. Familial interstitial pneumonia is the inherited form of interstitial pneumonia and is diagnosed in multiple members of the family whereas sporadic IPF affects only one member of the family. Research on the genetic makeup of sporadic IPF and the currently recognized mutations related to familial form of IPF emphasizes the significance of the lung epithelium in the progression of the disease [9]. Mutations in the genes involved in maintaining telomere length including telomerase reverse transcriptase (TERT), telomerase RNA component (TERC), (Fe-S) cluster containing regulator of telomere elongation helicase (RTEL1), poly-A specific ribonuclease (PARN), nuclear assembly factor 1 ribonucleoprotein (NAF1), TERF1 interacting nuclear factor2 (TINF2) and dyskerin (DKC1) have been discovered in approximately 25–30% of the familial IPF [37–42]. The mutations in these genes result in short telomeres, which lead to AEC2 senescence [43]. Mutations in desmoplakin (DSP), A-kinase anchoring protein 13 (AKAP13), catenin alpha 1 (CTNNA) involving in the epithelial cell integrity have also been identified in IPF [44–46]. Mutations in the gene for SFTPC, which is specifically expressed by AEC2, lead to dysfunctional surfactant folding and processing, ER stress, deregulated proteostasis, and possibly epithelial–mesenchymal transition [47]. Polymorphism of MUC5B (a gene associated with mucociliary clearance) is considered as one of the most prominent genetic factors in IPF [48]. Moreover, mutations in the toll interacting protein (TOLLIP), oligonucleotide/oligosaccharide binding fold containing 1 (OBFC1), TERT and TERC genes have been involved in the sporadic IPF [9].

##### Microorganisms

Epstein-Barr virus has been isolated from IPF lung epithelia [49]. Human herpes virus (HHV) is found to co-localize with the markers of ER stress and unfolded protein response (UPR) in AEC2 [50]. HHV can cause the disease by causing mutations in SFTPC and AEC2 dysfunction, which ultimately leads to ER stress and UPR [50]. Several bacteria were observed in the lungs of IPF patients, including pathogenic gram-positive bacteria such as *Staphylococcus sp* and *Streptococcus sp* [51]. The dysbiosis in the lung has been linked to the clinical markers of disease progression [52].

### Pathophysiology of IPF

**Fig. 1:**
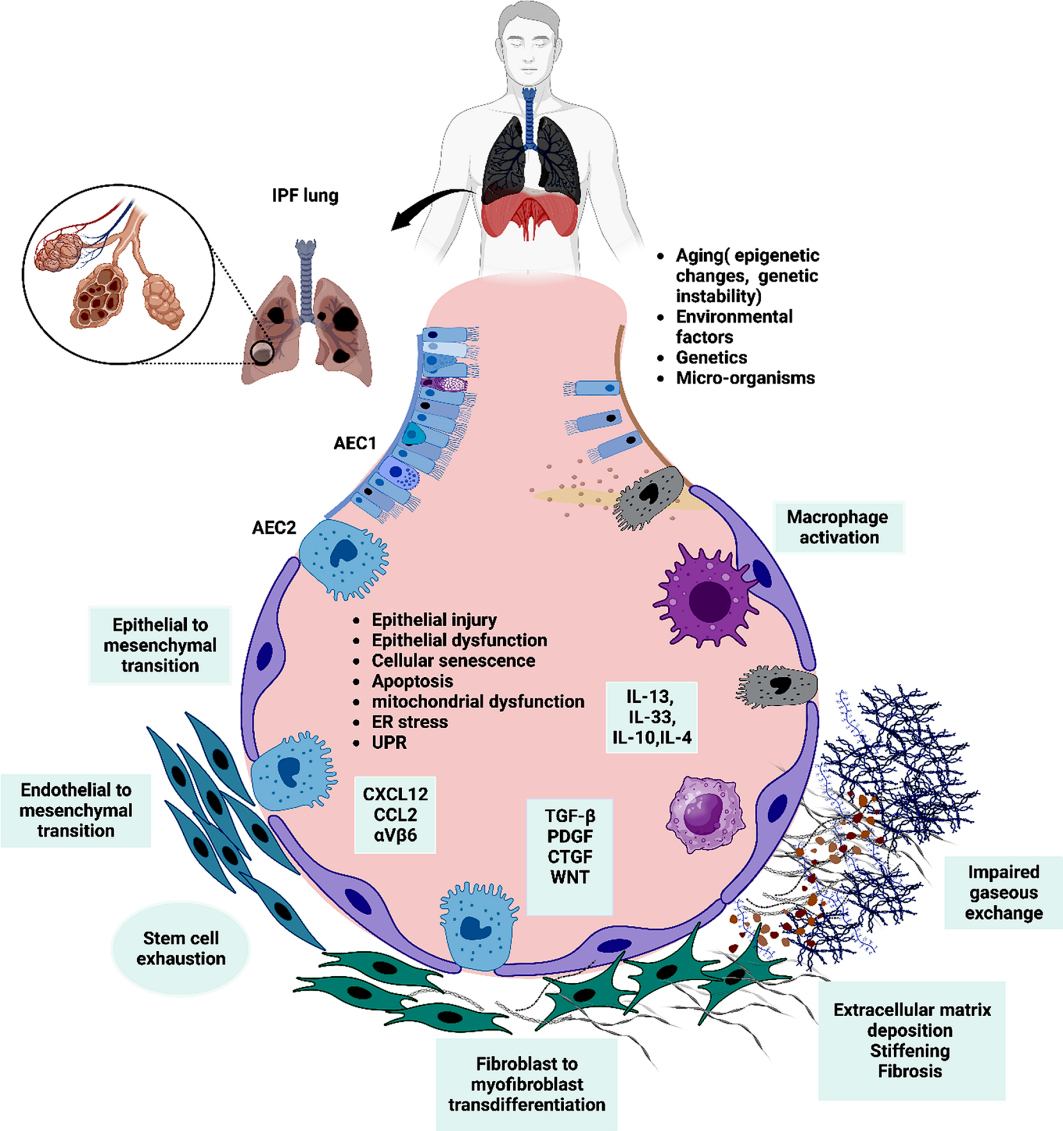
A model of pathophysiology of IPF. A synergistic effect of Aging, environmental factors, genetic makeup, and microorganisms elicit an epigenetic reprogramming, resulting in alveolar epithelial cell injury and stem cell exhaustion. Alveolar dysfunction and abnormal activation of alveolar epithelial type 2 cells (AEC2) occur as sequalae to this injury. The activated AEC2 secrete profibrotic cytokines (TGFβ, PDGF, WNT and CTGF) and chemokines (CXCL2 and CCL2), which recruit migrating fibrocytes and fibroblasts and result in the activation and transdifferentiation of fibroblasts into myofibroblasts. Myofibroblasts produce excess extracellular matrix which occupies the interstitial spaces, causing mechanical stiffness, remodelling of pulmonary architecture and fibrosis. Activated macrophages also help in the fibrosis process by becoming the source of pro-fibrotic molecules and enhance fibroblast proliferation. AEC2 and endothelial cells undergo epithelial and endothelial to mesenchymal transition, contributing to pro-fibrotic cells (Created with BioRender.com)

While an established cause is absent for the initiation of IPF pathogenesis, it has been widely accepted that repeated microinjuries to the alveolar epithelium in a genetically susceptible individual initiate an abnormal reparative process, which ultimately results in fibrosis (Fig. 1). The aberrantly activated AEC2 following lung injury secrete profibrotic cytokines, such as Transforming Growth Factor-β (TGF-β), platelet derived growth factor (PDGF), and connective tissue growth factor (CTGF), and chemokines, such as C-X-C motif chemokine ligand 2 (CXCL2) and C-C motif chemokine ligand 2 (CCL2) [53–56]. AEC2 are also responsible for a pro-fibrotic feedback loop through the activation of Wnt pathway which crosstalk with TGF-β [57].

Under the influence of TGF-β, the epithelial cells undergo epithelial to mesenchymal transition, where the epithelial cells acquire the features of mesenchymal cells. For instance they lose their polarity and become mobile. This is characterized by the upregulation of alpha-smooth muscle actin (α-SMA) and downregulation of E-cadherin and syndecan [58, 59]. In addition to EMT, endothelial to mesenchymal transition (EndoMT) has also been reported [60, 61].

The release of TGF-β is one of the major pro-fibrotic factors which promotes the differentiation of fibroblasts to myofibroblasts [62]. Apart from TGF-β, epithelial cells can also secrete Wnt proteins, which activate fibroblasts through Wnt signalling [63]. These fibroblasts are characterized by having phenotype that can resist apoptosis, along with high proliferation potential [64], whereas myofibroblasts are characterized by the high expression of α-SMA and can produce extracellular matrix (ECM) proteins such as type 1 and type 3 collagen [65]. The deposition of extracellular matrix into the interstitial space for a chronic period causes stiffening and gradual lung remodeling. Moreover, it was proposed that ECM can signal the mesenchymal cells to release additional ECM, resulting in an amplified loop of matrix production and deposition [66].

## Circrnas

### History of circRNAs

CircRNAs were first described while studying potato spindle tuber disease in 1971 and subsequently in plant viroids in 1976 by Sanger et al who defined it as covalently closed structures [67, 68]. A decade later, circRNAs were identified in human hepatitis delta virus [69]. They were initially thought to be yielded from the “mis-splicing” of exons or introns [70]. Later studies have concluded that circRNAs are covalently closed ring-like structures without 5’ or 3’ polarity or a polyadenylated tail and are formed by a type of alternative splicing called back-splicing during co-transcriptional and post transcriptional processes. Back-splicing takes place when a downstream 5’ splice site joins with an upstream 3’ splice site. This is contrary to the conventional linear splicing in which an upstream 5’ splice site ligates with a downstream 3’ splice site. The canonical pre-mRNA splicing results in a linear RNA whereas back-splicing generates a circular RNA molecule containing single or multiple exons [71].

In 2012, Salzman et al. found circRNAs in normal and malignant human cells and described them as RNA transcripts which are not arranged in a canonical order [72]. In 2013, Memczak et al. showed that circRNAs can function as post transcriptional regulators. For example, a circCDR1 functions as a sponge for miR-7 [5]. A study using cryo-electron microscopy to examine structures of the yeast spliceosomal E complex by Li et al. uncovered that canonical spliceosome is essential for back-splicing of circRNAs [73]. In 2017, Piwecka et al. have performed a circRNA knockout study involving circCDR1 in mice to investigate the interactions between circRNA and miRNA in the brain [74]. Various investigations have unearthed the diverse functions of circRNAs in humans and other organisms in normal and pathological conditions which are discussed later in this article.

### Types of circRNAs

CircRNAs can be classified based on their origin [75]. (1) Exon-only circRNA: Most of the highly expressed circRNAs consist of multiple exons from the pre-mRNA. Exon only circRNAs are located predominantly in the cytoplasm [76]. (2) Intron-only circRNA: The biogenesis for this class of circRNAs occurs due to the failure in debranching and depends on RNA motifs near 5’ splice site and branch point. They can regulate their parenteral gene expression by modulating the polymerase II activity. They are in the nucleus and have less miRNA binding sites compared to exon only circRNAs [77]. (3) Both intron and exon containing circRNAs: The introns are retained between the exons during the circularization process for this class of circRNAs. They can form complexes with U1 small nuclear ribonucleoprotein (snRNP) and interact with polymerase II transcription complexes to enhance gene expression of their parenteral gene. Like the intron only circRNAs, they predominantly reside in the nucleus [78]. (4) CircRNAs from fusion gene: They arise from cancer associated chromosomal translocations. Their function is to promote tumor cell survivability and transformation, thereby providing resistance against the anti-neoplastic treatment [77].

### Biogenesis of circRNAs

**Fig. 2:**
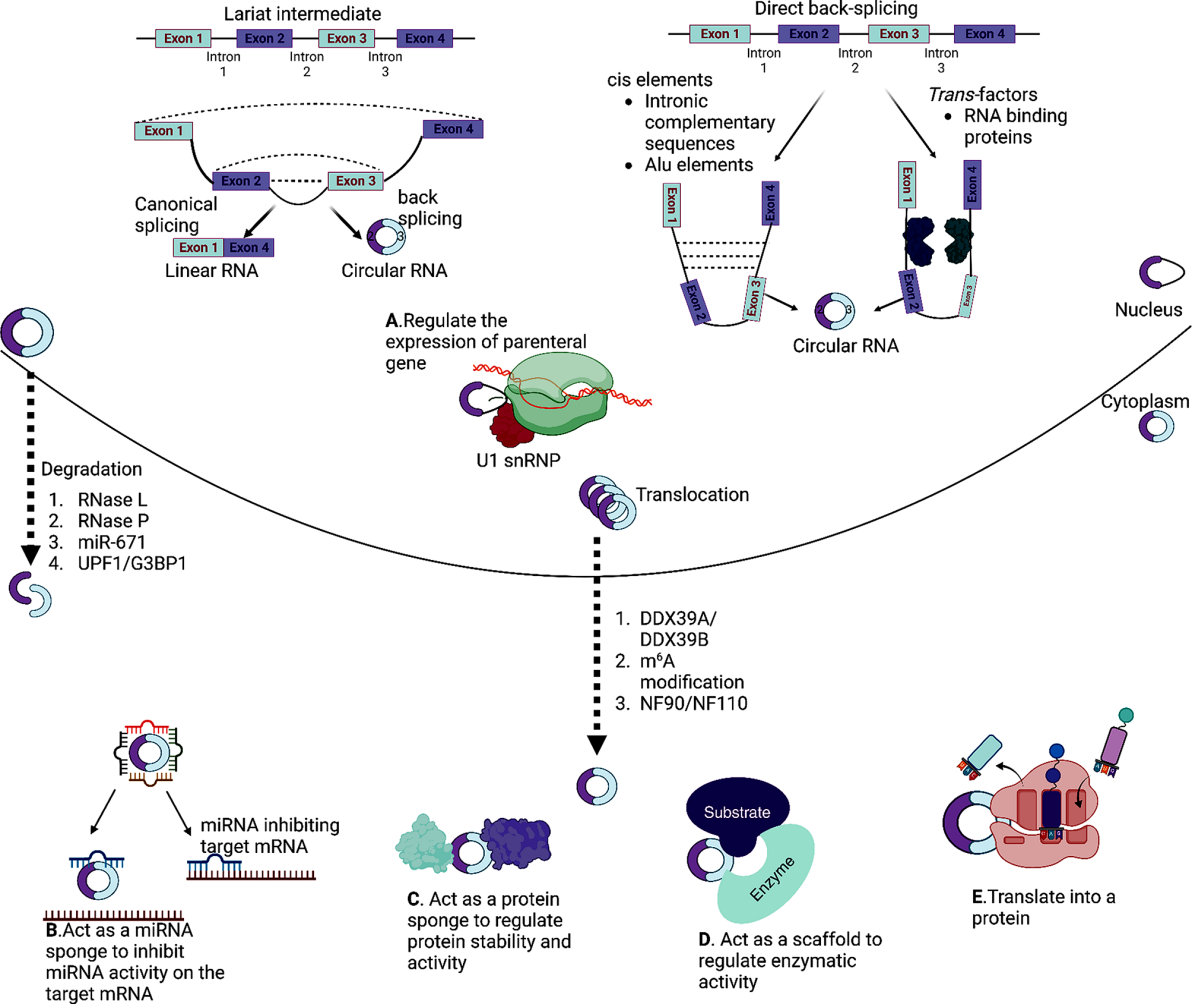
Biogenesis, trafficking, degradation, and biological functions of circRNAs. CircRNAs arise from pre-mRNA by lariat intermediate and direct back-splicing methods. After biogenesis, circRNAs are transported into cytoplasm by DDX39B/DDX39A, NF-90/NF-110 or through m^6^A. CircRNAs can undergo degradation in the cytoplasm upon the endonucleolytic cleavage endonucleolytic cleavage by RNase P, RNase L and miR-671 or structure-mediated degradation by UPF1 and G3BP1. CircRNAs can function as (**A**)- regulator of parenteral gene expression, (**B**)- miRNA sponge, (**C**)- protein sponge, (**D**) -protein scaffold, and (**E**)- template for translation. (Created with BioRender.com)

CircRNAs are generated from pre-mRNA and the circularization occurs through back-splicing. Back-splicing is often coupled with canonical splicing and uses canonical spliceosome machinery [79]. There are predominantly two proposed models for circRNA biogenesis naming “direct back-splicing” or “lariat intermediate” (Fig. 2). In direct back-splicing model, the back-splicing leads to the formation of a circRNA and a linear intermediate containing exons and introns, which can either be degraded or undergone canonical splicing, resulting in a linear RNA with skipped exons. In the lariat intermediate model, the canonical splicing occurs first to generate a linear RNA with skipped exons and a lariat consisting of exons and introns, which further undergoes back-splicing to form a circRNA [80].

*Cis*-elements and *trans* factors can promote back-splicing by bringing the 3’ donor and 5’acceptor site together [81]. Exon circularization can be achieved by forming RNA pairing between complementary base pairs across flanking introns. Among flanking introns, intronic complementary sequences having as low as 30–40 nucleotides are sufficient to facilitate circRNA formation [82]. In humans, back-splicing through *cis* elements includes base pairing between repeated complementary *Alu* elements [83] as well as non-repetitive complementary sequences [79]. Interestingly, the importance of complementary base pairing often is limited to mammalian genes which are rich in repetitive elements like *Alu* elements [84].

*Trans* factors that participate in circRNA biogenesis include certain proteins involved in the spliceosome functions and RNA binding proteins (RBPs). A study conducted in *Drosophil*a cells by Liang et al showed that upon depletion of the spliceosomal factors such as U1 and U2 snRNPs, the circRNA expression was elevated with a reduction in the linear mRNA synthesis [85]. U1 snRNP is required for the selection of the pre-mRNA at splice sites and U2 snRNP binds to the branch sites to facilitate canonical splicing [86]. RBPs can promote or repress circRNA biogenesis by binding complementary sequences in the flanking introns or by directly uniting 3’ to 5’ splice sites. RNA binding proteins muscleblind (MBNL1) and quaking (QKI) promote circularization, while RNA editing enzyme adenosine deaminase acting on RNA 1 (ADARI1) and DExH-box helicase 9 (DHX9) which possess the double stranded RNA binding domains are reported to reduce circularization [87–90]. RBPs such as nuclear factor 90 (NF90) and NF110 promote circRNA formation during viral infections [91]. Additional RNA binding proteins such as Fused in Sarcoma (FUS), nudix hydrolase 21 (NUDT21), and neurotumor ventral antigen 2 (NOVA2) also promote circRNA formation [92–94].

### CircRNA trafficking, localization, and degradation

Even though circRNA biogenesis happens in the nucleus, most of the exons only circRNAs are localized in the cytoplasm. Only a few circRNAs which contain introns are located in the nucleus [95]. CircRNAs are exported into the cytoplasm using RNA helicase and its length determines which protein to use. Two of these helicases are UAP56 (DDX39B) and URH49 (DDX39A) in *Drosophila melanogaster* or two homologues of Hel25E in humans. The depletion of DDX39B leads to the nuclear retention of long circRNAs with more than 1,300 nucleotides, whereas the depletion of DDX39A results in the accumulation of short circRNA with less than 400 nucleotides in length [96]. N6-methyladenosine (m^6^A) modification also takes part in the translocation of circRNAs. A recent study shows that m^6^A of circNSUN2 enhances its cytoplasmic export [97].

CircRNAs can also be secreted into extracellular space via exosomes (extracellular vesicles), but the exact mechanism of this delivery and translocations are unknown [98]. In a study conducted on pancreatic ductal adenocarcinoma, Circ-PDE8A was found in exosomes secreted by tumor cells into the blood circulation [99]. CircRNA-SORE and circPTGR1 can be transferred from more malignant cancer cells to less malignant cancer cells to elevate the malignancy potential via exosomes [100, 101].

The degradation of mRNAs occurs due to poly A-tail shortening by deadenylase, followed by decapping and finally decay by exoribonuclease. However, due to the lack of poly A-tail and 5’ 7- methylguanosine cap, circRNAs are cleaved internally by an endonuclease including RNase P, RNase L and miR-671. CircRNAs containing m^6^A recruit adapter protein HRSP12, which bring m^6^A reader protein YTHDF2 and exoribonuclease RNase P/MRP together, resulting in the rapid degradation of circRNAs [102]. Some circRNAs can form RNA duplexes and inhibit dsRNA-activated protein kinase associated with innate immunity responses. These circRNAs are degraded by RNase L during viral infection [103]. CircRNAs can also undergo degradation via miRNAs. miR-671-loading Ago2 is recruited to the miR-671 binding site of circCDR1, leading to the Ago2-mediated endonucleolytic cleavage, followed by exonucleolytic activity [104]. Other RNA binding proteins such UPF1 and G3BP1 can also participate in the degradation of circRNAs via structure-mediated RNA decay [105].

### Mechanisms of circRNA action

CircRNAs are understudied among other non-coding RNAs and the investigations on their biological functions are limited. Due to the unique structural features of circRNAs, they can offer binding sites to miRNA and RBPs and regulate their respective target genes. CircRNAs can function as a miRNA sponge and regulate the expression of miRNA target genes. Similarly, circRNAs also act as a protein sponge. Other functions of circRNAs in protein binding include acting as protein scaffolds and recruitment of proteins to specific locations. circRNAs have been reported to play a role in the transcriptional regulation of its parenteral gene. CircRNAs can also be translated into proteins.

The most well-known and studied function of circRNAs is its miRNA sponge-like activity. CircRNAs can have multiple binding sites for miRNAs and act as their sponges. When circRNAs reach the cytoplasm, they function as competing endogenous RNAs (ceRNAs) that bind with miRNAs and thereby inhibit the miRNA’s action on the target genes [106]. Therefore, indirectly, circRNAs play a role in gene regulation and alter the course of various cellular events such as cell proliferation, migration, metastasis, and apoptosis. Cerebellar degeneration-related protein 1 transcript (CDR1as) is one of the widely known circRNAs, which can function as a miRNA sponge with 63 known binding sites for miR-7 [5].

CircRNAs also possess binding sites for various proteins and can function as sponge, scaffold and decoys of proteins [107]. CircRNAs participate in the regulation of protein expression via their protein sponge activity. For example, CircPABPN1 suppresses the translation of its host gene PABPN1 and reduces cellular proliferation by acting as a sponge or decoy for human antigen R (HuR) [108]. This study provides a good example of how a circRNA competes with its parenteral mRNA for an RBP that affects translation. Similarly, cia-cGAS act as a sponge for nuclear cGAS and thereby inhibits its enzymatic activity to avoid cGAS-mediated exhaustion of dormant long term hematopoietic stem cells (LT-HSCs) [109].

Circ-Amotl1 can enhance the survival of cardiomyocytes because of its protein scaffold activity by binding with pyruvate dehydrogenase kinase 1 (PDK1) and serine-threonine protein kinase AKT1 [110]. Circ-Foxo3 binds to cyclin-dependent kinase (CDK2) and cyclin-dependent kinase inhibitor 1 (p21) to form a ternary complex and cause the repression of cell cycle progression and proliferation in non-cancerous cells. CDK2 is involved in cell cycle progression and upon forming the ternary complex with circ-Foxo3, the function of CDK2 is lost [111]. Similarly, circACC1 functions as a protein scaffold by enhancing the 5’ AMP-activated protein kinase (AMPK) through the assembly of a ternary complex with regulatory β and γ subunits [112].

Double-stranded binding proteins such as NF-90 and NF-110 take part in the biogenesis of circRNAs [91]. On the other hand, circRNAs can form imperfect intramolecular ds-RNA. Hence, circRNAs can suppress NF-90 and NF-110 which are ds-RNA receptors under normal conditions. During a viral infection, the action of endonuclease RNase L causes circRNAs to be degraded, thereby releasing NF-90 and NF-110 to facilitate an antiviral immune response [103]. Thus, the degradation of circRNAs can play a significant role in the immune response after viral infection by interacting with RNA binding proteins. CircRNAs have also been reported to interact with activated protein kinase to promote immune response in the event of viral infections [103].

CircRNAs located in the nucleus normally function as transcriptional regulators. Intron-containing circRNAs interact with U1 snRNP to promote the transcription of their parenteral gene [78]. For example, exon-intron circRNAs, circPAIP2 and circEIF3J and intron only circRNA, ci-ankrd52 regulate the expression of its parenteral genes by enhancing RNA polymerase II activity [78, 113]. CircSEP3 in thaliana *sp* forms an R-loop with its cognate DNA and leads to transcriptional pausing, which in turn increases splicing of the cognate exon 6-skipped variant of SEP3 gene [114].

Even though the mechanism and regulation of circRNA translation is not fully understood, strong evidence suggests a high possibility that some circRNAs can undergo translation. Endogenous circRNAs containing internal ribosomal entry site (IRES) and m^6^A as well as certain artificial circRNAs can be translatated [115–117]. CircZNF609 serves as an illustration of a protein-coding circRNA in eukaryotes and is translated into a protein in a splicing-dependent and cap-independent manner [118]. Circ-ZNF609 has an open reading frame starting at the start codon and ending at an in-frame STOP codon that results from circularization. CircZNF609 participates in the proliferation of myoblasts in Duchenne muscular dystrophy. A few circRNAs that have the ability to translate are found to be involved in the process of tumor suppression. In human glioblastoma, SHPRH-146aa, which was produced from the overlapping genetic codes of circ-SHPRH, acts as a tumor suppressor [119]. circ-FBXW7 having internal ribosomal entry site encodes for protein FBXW7-185aa. The cancer cell proliferation is inhibited when FBXW7-185aa is upregulated in vitro and in vivo [120].

## Regulatory roles of circrnas in the pathogenesis of IPF

CircRNA expression profiling in various pulmonary fibrosis models using RNA sequencing, microarray analysis and bioinformatics analysis has been tremendously useful in the identification of dysregulated circRNAs [121, 122]. So far, few circRNAs having a functional role in IPF have been identified. These circRNAs have either pro-fibrotic or anti-fibrotic effects. Their specific target genes and functions via various molecular mechanisms have been studied. Table 1 shows a list of dysregulated circRNAs whose functional roles in pulmonary fibrosis are known. We discuss below these circRNAs based on cell types they act on.

**Table 1:** Dysregulated circRNAs in pulmonary fibrosis and their mechanisms of action

Name	Cell type	Mechanism of action	Target genes and pathways	Effect	In vivo model	Reference
CircHIPK3	Fibroblasts (MRC-5, NIH-3T3, mouse primary lung fibroblasts)	miRNA sponge	miR-3a-3p & FOXK2 Glycolysis	Fibroblast-myofibroblast differentiation	SiO2-induced mouse lung fibrosis	[123]
Circ0044226	Fibroblasts (WI-38, HPF)	miRNA sponge	miR-7 & SP1 TGFβ1 signaling	Fibroblast-myofibroblast differentiation	Bleomycin-induced mouse lung fibrosis	[124]
CircHIPK3	Fibroblasts (WI-38)	miRNA sponge	miR-338-3p & SOX4, COL1A1	Fibroblast-myofibroblast differentiation	Bleomycin-induced mouse lung fibrosis	[125]
CircANKRD42	Fibroblasts (MRC-5)	miRNA sponge	miR-324-5p & YAP1 miR-136-5p & AJUBA YAP1 signaling and mechanical stiffness	Fibroblast-myofibroblast differentiation	Bleomycin-induced mouse lung fibrosis and IPF blood	[126]
Circ-949 and Circ-057	Fibroblasts (L929)	miRNA sponge	miR-29b-2-5p	Fibroblast proliferation & activation	Bleomycin-induced mouse lung fibrosis	[121]
CircHIPK2	Fibroblasts (HPF-α)	miRNA sponge	miR-506-3p	Fibroblast activation	SiO2-induced mouse lung fibrosis	[127]
CircTADA2A	Fibroblasts (Normal and IPF HPF)	miRNA sponge	miR-526b & Cav-1 miR-203 & Cav-2	Fibroblast-myofibroblast differentiation	Bleomycin-induced mouse lung fibrosis	[128]
CircSPON1	Fibroblasts (Human fetal lung fibroblasts)	miRNA sponge	miR-942-5p/ miR-52f-3p & Smad-7	Fibroblast activation	Bleomycin- induced mouse lung fibrosis	[129]
CircHECTD1	Fibroblasts (HPF)	unknown	HECTD1 Autophagy	Fibroblast activation	SiO2-induced mouse lung fibrosis	[130]
Circ-012091	Fibroblasts (I-929 and HPF)	unknown	PPP1R1B	Fibroblast proliferation & migration	SiO2-induced mouse lung fibrosis	[131]
CircRNA-662 CircRNA-949	Fibroblasts (L929)	miRNA sponge	miR-29b & Gli2/STAT3	Fibroblast activation	Bleomycin- induced mouse lung fibrosis	[132]
Circ0000672 and Circ0005654	Fibroblasts	Protein scaffold	elF4A3	Fibroblast dysfunction	SiO2-induced mouse lung fibrosis	[133]
Circ0026344	Fibroblasts (MRC-5)	miRNA sponge	miR-21	Fibroblasts activation	Cigarette smoke-induced mouse lung fibrosis	[134]
CircZC3H4	Epithelial cells (MLE12)	miRNA sponge	miR-212 & ZC3H4 ER stress	Epithelial to mesenchymal transition	SiO2-induced mouse lung fibrosis	[135]
Circ0044226	Epithelial cells (RLE-6TN)	Protein sponge	CDC27	Epithelial to mesenchymal transition		[136]
CircCDR1	Epithelial cells (Pulmonary epithelial cells)	miRNA sponge	miR-7 & TGFBR2	Epithelial to mesenchymal transition		[137]
Circ0000981	Epithelial cells (Mouse lung TC-1)	miRNA sponge	miR-211-5p & TGFBR2	Epithelial to mesenchymal transition	OVA (asthma)-induced pulmonary fibrosis	[138]
CircHECTD1	Endothelial cells (MML1, HUVEC)	Protein sponge	HECTD1	Endothelial mesenchymal transition	SiO2-induced mouse lung fibrosis	[61]
CircHECTD1	Macrophage (RAW264.7)	Protein sponge	HECTD1 ZC3H12A ubiquitination	Macrophage activation	SiO2-induced mouse lung fibrosis	[139]
CircZC3H4	Macrophages (RAW 264.7)	Protein scaffold	ZC3H4	Macrophage activation	SiO2-induced mouse lung fibrosis	[140]
circRNA11:120406118|12,040,782	Macrophages (THP-1)	miRNA sponge	miR-30b-5p & NLRP3 inflammasomes & pyroptosis	Macrophage activation & pyroptosis	SiO2-induced mouse lung fibrosis	[141]
CircPWWP2A	Macrophages Fibroblasts (RAW 264.7, NIH/373)	miRNA sponge	miR-223-3p & NLRP3	Macrophage activation	SiO2-induced mouse lung fibrosis	[142]

### Fibroblasts

Fibroblast activation and differentiation of fibroblasts to myofibroblasts are unmistakably the pivotal pathological process, which ultimately leads to the development of lung fibrosis. Several circRNAs are reported to be involved in this process.

#### Upregulated circRNAs in fibroblasts

Previous studies have shown that increased glycolysis promotes lung fibrosis by stabilizing HIF-1α, which facilitates the differentiation of fibroblasts into myofibroblasts. The expression of glycolytic enzymes are increased in the fibrotic lungs and the inhibition of the glycolysis prevents the differentiation of fibroblasts to myofibroblasts [143]. A study by Xu et al. explored the effects of circHIPK3 on glycolysis and fibroblast activation [123]. They showed that circHIPK3 expression level was upregulated in TGFβ1-treated human pulmonary fibroblasts in vitro. The inhibition of circHIPK3 reduces the glycolysis and proliferation of pulmonary fibroblasts. The silencing of circHIPK3 in vivo using adeno-associated virus vector inhibits silica-induced lung fibrosis. The FOXK2 gene is a transcription factor involved in glycolysis and is inhibited by miR-30a-3p. The model proposed by Xu et al. suggests that circHIPK3 sponges the inhibitory action of miR-30a-3p and increases FOXK2 expression, resulting in enhanced glycolysis in fibroblasts and facilitating its activation. Another study showed a similar effect of circHIPK3 on the differentiation of fibroblasts to myofibroblasts by a different mechanism [125]. In this study, circHIPK3 acts as a sponge for miR-338-3p and thereby enhances the expression of SOX4 and COL1A1, which in turn facilitates the differentiation of fibroblasts to myofibroblasts.

In a study examining the roles of lung fibroblasts in silicosis, it is found that the treatment of human pulmonary fibroblasts with silica increases Sigma-1 receptor expression, which induces ER stress and promotes the migration and activation of fibroblasts [127]. circHIPK2 is also induced in pulmonary fibroblasts by silica. The induced circHIPK2 expression competes with miR-506-3p, leading to the increase in Sigma-1 receptor level and thus fibroblast activation.

Circ0044226 was found to be upregulated in bleomycin-treated mice and TGFβ1-treated pulmonary fibroblasts [124]. The knockdown of circ0044226 in vivo and in vitro inhibits fibroblast differentiation. The luciferase reporter assay reveals that Circ004226 sponges miR-7 and thus increases the expression of sp1, a target of miR-7 and a transcription factor for TGFβ1, suggesting that circ0044226 is a profibrotic factor in lung fibroblasts via competing with miR-7.

CircRNA-ankyrin repeat domain 42 (CircANKRD42) was identified as an upregulated circRNA from the peripheral blood of IPF patients [126]. CircANKRD42 is generated by reverse splicing activated by hnRNPL. CircANKRD42 promotes lung fibroblast migration and differentiation by facilitating the crosstalk between mechanical stiffness and biochemical signals through sponging of two different miRNAs. The sponging of miR-136-5p by circANKRD42 increases the expression of the miR-136-5p target, yes-associated protein 1 (YAP1). The sponging of miR-324-5p by the same circRNA elevates its target, ajuba LIM protein (AJUBA), which inhibits the binding of large tumor suppressor kinase 1/2 (LATS1/2) and p-YAP1 and results in enhanced nuclear translocation of YAP1. Elevated YAP1 levels in the nucleus as a result of sponging both miRNAs initiate transcription of the genes which causes mechanical stiffness such as F-actin and Myo1c.

#### Down-regulated circRNAs in fibroblasts

Apart from the above-mentioned upregulated circRNAs, a few circRNAs are downregulated in IPF and show antifibrotic functional characteristics.

CircTADA expression is reduced in IPF fibroblasts compared to normal lung fibroblasts [128]. CircTADA inhibits the proliferation and activation of human lung fibroblasts by sponging miR-526b and miR-203 and in turn elevating the expression of caveolin-1 and caveolin-2, respectively.

Circ949 and circ057 are upregulated in a mouse model of bleomycin-induced lung fibrosis [121]. Both circRNAs sponge miR-29b-2-5p. Although miR-29 mimic inhibits the proliferation and activation of fibroblasts, the effects of cicr949 and circ057 on fibroblast functions are unknown.

Protein phosphatase 1 regulatory subunit 13B (PPP1R13B) is a member of the p53 family that promotes apoptosis and is upregulated in the silica-induced pulmonary fibrosis model. CRISPR knockout of PPP1R13B inhibits silica-induced ER stress and autophagy as well as fibroblast proliferation and migration [131]. Circ012091 is downregulated in silica-treated lung fibroblasts and the lung tissue of silica-treated mice. The overexpression of circ012091 reduces PP1R13B expression. No studies were performed on the role of circ012091 on fibroblast functions.

Similarly, homologous to the E6-AP C-terminal domain E3 ubiquitin protein ligase 1 (HECTD1) is upregulated and circHECTD1 is downregulated in silica-exposed fibroblast cells [130]. HECTD1 mediates silica-induced lung fibroblast activation via autophagy. CircHECTD1 has an opposite effect on fibroblast function by reducing HECTD1 protein levels.

CircSPON1 generated from F-spondin 1 (SPON1) under the influence of forkhead box O3 (FOXO3) is involved in pulmonary fibrosis through the suppression of fibroblast activation by inhibiting the translocation of SMAD-3 into the nucleus. CircSPON1 also acts as a sponge for miR-942-5p and miR-520f-3p and increases the expression of SMAD-7 which regulates TGF-β signaling negatively [129]. Signal transducer and activator of transcription 3 (STAT-3) and zinc finger protein (Gli-2) are pro-fibrotic molecules which are involved in pulmonary fibrosis, and it was found that circRNA-662 and circRNA-949 have sponge like activity against miR-29b which interacts with STAT-3 and Gli-2. However, the exact mechanism of their function in pulmonary fibrosis remains unknown [132]. Circ0026344 is downregulated in mouse lung fibrosis induced by cigarette smoke extract, and it act as a sponge for miR-21 and the downregulation of Circ0026344 causes significant upregulation of exosomal miR-21, leading to the inhibition of smad-7. This activates an anomalous crosstalk between epithelial cells and fibroblasts. resulting in fibroblast activation [134].

#### m6A modified circRNAs in fibroblasts

m^6^A is a well conserved transcriptional modification in eukaryotic cells and is involved in the initiation and pathogenesis of human cancers [144]. Using m^6^A-epitranscriptomic microarray, two circRNAs, hsa_circ_0000672 and hsa_circ_0005654 were identified to undergo m^6^A modification in the lungs of a mouse model of silicosis [133]. The methyl transferase 3, N6-adenosine-methyltransferase complex catalytic subunit (METTL3) was identified to be responsible for m^6^A modification of these two circRNAs. Simultaneously knockdown of hsa_circ_0000672 and hsa_circ_0005654, but not individual circRNAs leads to fibroblast dysfunction. This effect appears due to their binding with eIF4A3 protein, a eukaryotic translation initiation factor. However, how the circRNAs affect the eIF4A3 activity was not studied.

### Epithelial cells

AEC2 serves as stem cells within lung tissues [145]. They play a crucial role in repairing and regenerating the lung’s epithelium following injuries and they have been implicated in the development of IPF. However, when AEC2 cells become dysfunctional or undergo apoptosis, this can lead to stem cell depletion, triggering abnormal and uncontrolled reparative processes that contribute to the formation of pulmonary fibrosis [146]. During fibrogenesis, AEC2 may undergo a transition called epithelial to mesenchymal transition (EMT), in which they lose their typical epithelial characteristics and adopt mesenchymal traits [147]. Although numerous studies show the involvement of EMT in IPF in vitro, the development of EMT in vivo remains a subject of controversy [148]. For example, in a study of bleomycin-induced mouse lung fibrosis, clear evidence for a complete transformation of epithelial cells into mesenchymal cells was lacking [149].

CircCDR1 is upregulated in silica-treated lung epithelial cells [137]. This circRNA promotes EMT by acting as a sponge of miR-7 and upregulates the miRNA target gene transforming growth factor beta receptor 2 (TGFBR2). Zinc finger CCCH-type containing 4 protein (ZC3H4) is a transcription factor that increases EMT via ER stress [135]. circZC3H4 is upregulated in silica-treated lung epithelial cells. circZC3H4 sponges the activity of miR-212 and regulates the expression of ZC3H4 protein. As previously discussed, circRNA0044226 is increased by TGFβ1 and promotes the activation of lung fibroblasts [124]. circRNA0044226 was found to be the most upregulated circRNA in the lung tissue of IPF patients [136]. In addition to its role in fibroblasts, circRNA0044226 also regulates EMT in lung epithelial cells. Knockdown of circRNA0044226 inhibits EMT by downregulating the expression of cell division cycle protein 27 (CDC27) [136].

A study conducted on asthma-induced pulmonary fibrosis concludes that circ0000981/miR-211-5p/TGFBR2 interaction plays a role in the fibrosis development [138]. Atracytlon is a naturally found drug in surinam cherry and has anti-inflammatory properties. Upon the treatment with atracytlon, ovalbumin-induced expression of circ0000981 and TGFBR2 were significantly reduced whereas miR-211-5p was upregulated. In vivo studies confirm that atracytlon inhibits TGFβ1-induced EMT.

### Endothelial cells

Like EMT, endothelial to mesenchymal transition (EndoMT) also plays a significant role in the development of fibrosis through the accumulation of extracellular matrix. While resident fibroblasts and bone marrow fibrocytes are recognized as a source of myofibroblasts, endothelial to mesenchymal transition is a relatively newly recognized transdifferentiation process enriching mesenchymal cell population [60]. Such a transition was observed in a study conducted in a mouse model of silicon dioxide-induced-lung fibrosis, and mouse and human endothelial cell lines MML1 and HUVECs treated with silicon dioxide. In both mouse and human cell models, circHECTD1 was found to be upregulated and HECTD1 protein was downregulated by the treatment of silicon dioxide [61]. It is proposed that circHECTD1 downregulates the protein HECTD1 and thereby promotes EndoMT and lung fibrosis. This finding was confirmed using tissue samples obtained from silicosis patients and silicon dioxide-treated mice.

### Macrophages

Along with endothelial cells and epithelial cells, immune cells are also engaged in the process of fibrosis development. The results from single cell RNA sequencing data suggest that monocyte-derived alveolar macrophages are localized near areas of epithelial injury and activated fibroblasts and drive lung fibrosis in an asbestos mouse model [150]. During tissue repair, monocytes can differentiate into either M1 or M2 macrophages depending on the cytokine availability. IFNγ and lipopolysaccharide (LPS) facilitate the differentiation into M1 phenotype (pro-inflammatory), while IL-4, IL-10 and IL-13 influence the differentiation of M2 macrophages (pro-fibrotic) [151]. Under the fibrotic conditions, the profibrotic cytokines favor M2 differentiation and thereby result in increased secretion of TGFβ, PDGF, FGF and VEGF. The profibrotic cytokines released by M2 macrophage causes fibroblast activation and trans-differentiation, thus have a direct effect on the extra cellular matrix accumulation and mechanical stiffening [152].

A study conducted in silicon dioxide-induced lung fibrosis of mice shows that the expression level of circHECTD1 is reduced, but the expression of its host gene HECTD1 was increased in the macrophages isolated from bronchoalveolar lavage fluid (BALF) 7- and 28-days post treatment [139]. HECTD1 regulates cell polarity by ubiquitinating key proteins. This study emphasizes the interaction between circHECTD1 and HECTD1, which mediates the macrophage polarization through ZC3H12A ubiquitination. The circZC3H4/ZC3H4 pathway plays a role in macrophage activation in silicon dioxide-induced lung fibrosis mouse model and in macrophage cell line RAW264.7 [140]. Exosomal circRNA11:120406118|12,040,782 was found to be abundant in the peripheral serum of patients with silicosis. CircRNA11:120406118|12,040,782 is involved in the upregulation of NLR family pyrin domain containing 3 (NLPR3) by sponging miR-30b-5p. NLPR3 inflammasome is a crucial element of immune system that regulates caspase-dependent pro-inflammatory cytokine release and pyroptotic cell death in the advent of cell injury. By the inhibition of circRNA11:120406118|12,040,782 or overexpression of miR-30b-5p, silica-induced pyroptosis in macrophages under the influence of NLPR3 was alleviated [141].

circ002676 is involved in macrophage polarization in the SiO2-induced model of pulmonary fibrosis. Knockdown of circ002676 inhibited the expression of M2 macrophages, suggesting that this circRNA has a role in the M2 polarization of pulmonary macrophages. However, further studies are required to uncover the exact mechanism by which circ002676 regulates M2 polarization [153].

CircPWWP2A is a profibrotic circRNA sponging miR − 223–3p, which has an inhibitory effect on NLRP3 to promote pulmonary fibrosis. In SiO2-induced lung fibrosis, the CircPWWP2A/miR − 223–3p/ NLRP3 pathway has potential roles in the regulation of inflammation and fibrogenesis, hence highlighting its therapeutic significance [142].

## Application of circRNAs in diagnosis and therapy of IPF

### Detection methods of circRNAs

Unlike their linear counterparts, endogenous circRNAs are generally less abundant, which makes them difficult to detect [95]. Moreover, the traditional RNA detection methods using poly A tail fall short while detecting circRNAs due to its absence of poly A tail. Hybridization-based methods including Northern blotting, fluorescence in situ hybridization, microarray and amplification-based detection methods including RNA sequencing, real-time PCR, rolling cycle amplification (RCA), loop-mediated isothermal amplification (LAMP) are employed in circRNA detection. We briefly discuss microarray, RNA sequencing and real-time PCR.

#### Microarray

Microarray is a high throughput tool that can be used in the large-scale assessment of differentially expressed genes [154]. The difference in the microarray between linear RNAs and circRNAs is the design of the probe sequence [155]. CircRNA microarrays target the back-splicing junctions in circRNAs, and disregard linear RNAs since they are devoid of back-slicing junctions [156]. The disadvantage of this technique is that it cannot detect low abundance molecules.

#### Next-generation RNA sequencing

RNA sequencing allows the complete sequencing of RNAs from a tissue or cells. The development of RNA sequencing technology is unarguably the turning point which has made the genome-wide studies of circRNAs possible. Total RNA sequencing instead of mRNA sequencing is normally used for circRNAs as circRNAs do not have poly A tails. Ribosomal RNAs are depleted from total RNAs to enhance the sensitivity [157]. RNA sequencing for circRNAs adapts some methods to discriminate other RNAs from circRNAs, such as employing deeper sequencing with longer reads, and RNA exonuclease-based enrichment that eliminates linear RNAs [158]. A wide variety of software and identification tools are developed to analyze circRNAs from the RNA sequencing data such as CIRI, circ RNA finder, CIRC explorer, find CIRC, and UROBORUS etc. [159, 160]. The algorithms are designed in such a way to read through known splice sites in reverse order to identify a circular sequence. The most advantageous feature of RNA sequencing technology is that it can discover new circRNAs with high accuracy.

#### Real-time PCR

This method is considered as a gold standard test for the quantification of circRNAs and generally opts for validating the circRNAs identified through high through-put methods such as microarray and RNA sequencing [161]. Real-time PCR can be easily employed in any settings whether it is a clinical or research laboratory, making it the most user friendly among the circRNA detection tools. The main disadvantage of this method is that it has low throughput compared to the other methods. Also, in rolling circle amplification during reverse transcription, there might be a chance of formation of concatemers and this could hamper the accuracy of the data obtained [162].

### CircRNAs as a diagnostic tool

IPF is considered as an underdiagnosed disease. The ATS/ERS/JRS/ALAT has developed diagnostic criteria of for IPF [163]. These criteria require the patient to undergo high resolution CT (HRCT) along with a surgical lung biopsy. The technique of tracheobronchial lung cryobiopsy (TBLC) has also been introduced for a diagnosis of IPF [164]. The main disadvantage of these criteria is that it requires the patient to undergo tedious invasive surgical biopsies under general anesthesia.

Non-invasive methods such as using biomarkers are still not available. CircRNAs are highly stable and resistant to exonucleases or RNase R and possess a unique expression pattern. Their high abundance in the body fluids makes them an ideal candidate for non-invasive diagnostic methods. CircRNAs are detected in whole blood, individual blood cells (red blood cells, white blood cells and platelets) and serum [165]. circANKRD42 which is upregulated in the blood of IPF patients serves as an example of a potential biomarker [126]. In a study conducted by examining the plasma levels of IPF patients, 67 circRNAs were found to be dysregulated. Among them, hsacircRNA_100906, hsacircRNA_102100 and hsacircRNA_102348 were significantly upregulated depicting their biomarker potential [166].

CircRNAs are also abundant in exosomes. Exosomal circRNAs are already in the development as a biomarker in the diagnosis of cancers [167]. Exosomal circ-PDE8A can be used in the prediction of prognosis of pancreatic ductal adenocarcinoma [168]. In patients with colorectal cancer, hsa-circ-0004771 was found to be significantly upregulated and hence can be utilized as a biomarker [169]. Abundant levels of exosomal circRNA11:120406118|12,040,782 was reported in patients suffering from pulmonary fibrosis induced by silica exposure [141]. The most advantage in using circRNAs as diagnostic biomarkers for IPF is that it is a non-invasive simple procedure of blood withdrawal. However, the current technologies in detection of circRNAs are not economically feasible in clinical laboratory settings.

### CircRNAs as a therapeutic tool

As discussed earlier, circRNAs are involved in many pathological pathways, which lead to the development and progression of IPF. Hence, circRNAs can be targeted as a promising therapeutic. The approach will be either overexpressing anti-fibrotic circRNAs or knockdown of pro-fibrotic circRNAs. Knockdown of circRNAs can be achieved by RNA interference through short interfering RNA (siRNA) or short hairpin RNA (shRNA) [170, 171]. Lipid-based polymer delivery of siRNA and shRNA can be employed for in vivo circRNA knockdown [172]. Another method is CRISPR Cas-9-mediated deletion of exonic or intronic sequences [173]. CircRNA expression vectors such as lentiviral and adenoviral vectors can be used to overexpress a circRNA in vivo. CircRNA overexpression can also be made possible by using synthetic circRNAs exogenously [174, 175].

Ongoing studies also highlight the importance of efficient circRNA delivery systems. CircRNA delivery using nanoparticles has gained some attention in recent times. Utilizing gold nanoparticles (AuNPs) linked with siRNA that target circDnmt1 emerges as a potential therapeutic strategy for breast cancer [176]. One disadvantage of this method is that it could trigger an immune response in the host. Recently exosomes are being employed as vehicles for the expression of circRNA vectors. The administration of modified exosomes containing engineered rabies virus glycoprotein-circSCMH1 facilitates recovery in mice and monkeys suffered from ischemic stroke [177]. The advantage of using exosome as a delivery system over synthetic nanoparticles is that it facilitates circRNA cellular uptake without setting off the host immune system. In IPF, we already discussed a few circRNAs, which have therapeutic potential. For example, circTADA2A is shown to alleviate lung fibrosis in bleomycin-induced mouse lung fibrosis upon overexpression. Knockdown of has-circ0044226 in bleomycin-induced pulmonary fibrosis mouse model attenuates lung fibrosis, highlighting its potential as a therapeutic target [124].

The potential for circRNAs to be utilized as therapeutic targets is highly promising and could significantly advance the field of gene therapy. However, it is important to acknowledge that the investigation of circRNAs is still primarily in the research phase. Given the current state of knowledge about circRNAs, the translation of these findings into clinical settings is likely to be a considerable distance away. Presently, the key obstacle involves the identification of suitable circRNAs capable of mitigating or reversing the disease condition. Furthermore, once the circRNA is identified, achieving efficient circRNA distribution and ensuring target specificity also appears quite challenging. Exogenously synthesized circRNAs could elicit certain immune responses in some individuals and hence pose an obstacle in using it in vivo [178].

## Conclusion

IPF represents a disease condition that severely impacts the patient’s quality of life. Its prognosis is believed to be worse than cancers. Existing medications for IPF have limited efficacy in slowing down disease progression and fail to bring about noticeable improvements in the patient’s condition. In this context, circRNAs emerge as promising therapeutic targets and non-invasive biomarkers. New technologies have facilitated the discovery of dysregulated circRNAs in IPF. However, there are still critical questions that remain unanswered. Most RNA sequencing analyses for circRNAs have been conducted using bulk sequencing, necessitating the adoption of single-cell RNA sequencing to identify dysregulated circRNAs at a single cell level. Additionally, only a few circRNAs have undergone functional studies, warranting further research to identify key circRNAs involved in different cells during IPF pathogenesis. Most circRNAs studied in IPF are related to miRNA sponges, but other mechanisms of circRNA action need further exploration. This includes investigating how circRNAs interact with DNA, RNA, and proteins. Furthermore, a clearer understanding of the molecular processes governing circRNA biogenesis and subcellular localization is necessary. Lastly, it is crucial to identify and conduct functional studies on exosomal circRNAs associated with IPF. Looking ahead, we anticipate that more comprehensive investigations into the role of circRNAs in IPF will shed light on the unanswered questions and provide valuable insights in the future.

## Data Availability

Not applicable.
